# Imaging- and Device-Based Congestion Assessment to Guide Chronic Heart Failure Management: A Systematic Review and Network Meta-Analysis

**DOI:** 10.3390/diagnostics16213595

**Published:** 2026-10-30

**Authors:** Michail Spanos, Dimitrios Raptis, Franco Joaquin Cossettini, Armend Balidemaj, Ashwin Ragupathi, Zainab Elahmadi, Joo Hee Park, Praveen Kumar Komminni, Ali Maaz, Pascal Ezerioha, Petras Lohana, Zainab Sabir, Jodi-Ann King, Christoforos Meliadis, Dimitrios Strepkos, Theodoros Vichos, Camillo Duarte Calderon, Qayum Shah, Tajamal Hayat, Lydia Giannakou, Rahul Kumar, Michail Kladas, Mallika Pradhan, Damianos G. Kokkinidis

**Affiliations:** 1 North Central Bronx Hospital/Jacobi Medical Center, NYC Health + Hospitals, Albert Einstein College of Medicine, Bronx, NY 10467, USAcossettf@nychhc.org (F.J.C.); armendbalidemaj1@gmail.com (A.B.); ashwinragu1996@gmail.com (A.R.); maaza1@nychhc.org (A.M.); drpetraslohana@gmail.com (P.L.); rbatra390@gmail.com (R.K.);; 2 Cardiovascular Research Center, Massachusetts General Hospital, Harvard Medical School, Boston, MA 02114, USA; 3 Department of Orthopaedic Surgery, University of California, San Francisco, CA 94143, USA; 4 Minneapolis Heart Institute, Abbott Northwestern Hospital, Minneapolis, MN 55407, USAvichos.theodoros@gmail.com (T.V.); 5 Minneapolis Heart Institute Foundation, Abbott Northwestern Hospital, Minneapolis, MN 55407, USA; 6 Section of Cardiovascular Medicine, Department of Internal Medicine, Yale School of Medicine, Yale University, Dana 3, New Haven, CT 06520, USA

**Keywords:** heart failure, congestion, network meta-analysis, pulmonary artery pressure monitoring, lung ultrasound, intrathoracic impedance, remote monitoring, HF hospitalization

## Abstract

**Background/Objectives**: Congestion drives most heart-failure (HF) hospitalizations, and several strategies now titrate therapy to a directly measured congestion signal rather than to symptoms; whether they differ by the signal measured has not been examined comparatively. **Methods**: We performed a systematic review and contrast-based network meta-analysis of randomized trials comparing imaging- or device-based congestion-triggered management with usual care. Thirteen chronic-phase trials formed a star network (no closed loops; rankings exploratory) of four strategies, pressure-guided (N1), lung ultrasound (N2), transthoracic lung impedance (N3), and cardiac-implantable-electronic-device (CIED) intrathoracic impedance (N5), with HF hospitalization as the primary outcome and six prespecified sensitivity analyses; four acute trials formed a gap map. **Results**: Only pressure-guided monitoring reduced HF hospitalization (0.72, 95% confidence interval 0.57–0.90), stable across sensitivities and matching external data. Lung ultrasound did not reduce hospitalization (0.72, 0.48–1.08) or mortality but reduced urgent visits (0.26, 0.13–0.50). The apparent CIED-impedance harm (1.12, 0.77–1.63) reflected an audible-alert trial and dissolved when early-terminated trials were excluded (0.87). Lung impedance gave the largest estimate (0.51, 0.31–0.83) but rested on one trial. No strategy tested in more than one trial reduced mortality. **Conclusions**: The congestion signal acted upon, and who receives the alert, track with the effect, although signal and workflow cannot be separated within trials; clinicians should match modality to the intended outcome, as these tools defer hospitalization, not death.

## 1. Introduction

Hospitalization for worsening heart failure is preceded, in most patients, by days to weeks of rising filling pressures and accumulating lung water before symptoms cross the threshold that prompts care [1,2]. This latency is the rationale for strategies that act on a measured index of congestion rather than waiting for dyspnea or weight gain. Over two decades, the following four physiologically distinct approaches have been tested in randomized trials: implantable pressure sensors (pulmonary-artery, left-atrial, or right-ventricular) [3,4,5,6,7,8,9], serial lung ultrasound to quantify B-lines [10,11,12,13,14], transthoracic bioimpedance to track lung water [15], and CIED-based intrathoracic impedance with automated alerts [16,17].

The physiological premise is well-established. In the weeks before admission, left-sided filling pressures rise first, followed by a measurable increase in lung water and only then by weight gain, dyspnea, and the decision to seek care. This “hemodynamic congestion precedes clinical congestion” sequence explains why strategies anchored to symptoms or daily weights have repeatedly failed to prevent hospitalization [18]: by the time the monitored variable moves, the window for outpatient up-titration of diuretics has largely closed. The four strategies examined here each attempt to intervene earlier in that sequence, but they sample different points along it, intracardiac pressure at the proximal end, lung water in the middle, and device-derived impedance as a composite surrogate, and they differ in who receives the signal and how the therapeutic response is triggered. These strategies are usually discussed as a single category, “remote monitoring” or “congestion-guided care”, and meta-analyses have tended either to pool them indiscriminately or to examine one modality in isolation [19,20]. Neither approach answers the question a clinician actually faces: among strategies that act on a direct congestion signal, does the type of signal matter for outcomes? Pooling different modalities obscures the possibility that a pressure sensor and an alert-driven impedance algorithm have opposite effects; examining one modality at a time downgrades the common comparator (usual care) that makes the strategies commensurable. The practical consequence is that clinicians lack a comparative, decision-relevant map of congestion-guided options. The existing literature either reports a single blended “remote monitoring” effect or evaluates one modality at a time, so the central question, whether the type of congestion signal changes outcomes, has not been answered, and the apparent contradictions across trials have not been reconciled. This study addresses that gap by organizing the randomized evidence around the diagnostic modality that triggers treatment, ranking the strategies against a shared usual-care reference, stress-testing every node with prespecified sensitivity analyses, and triangulating against external modality-specific benchmarks, so that a reader can see not only which strategy ranks where, but how robust each estimate is and which outcome it actually moves.

We therefore organized the evidence as a network anchored on usual care, with each active node defined by the physiological signal that triggers management. We deliberately excluded two adjacent classes whose mechanism is not a direct congestion measurement, natriuretic-peptide-guided titration (a neurohormonal-biomarker strategy) [21,22,23,24,25,26,27,28,29,30,31,32,33] and CIED multiparameter surveillance (in which the congestion component cannot be isolated) [34,35,36], because including them would violate the transitivity that a shared usual-care comparator requires. The objective was to estimate, descriptively rank, and stress-test the comparative effectiveness of direct congestion-guided strategies for HF hospitalization, with all-cause mortality and urgent HF visits as secondary outcomes, and to map the separate acute/in-hospital evidence that does not yet form a connectable network.

## 2. Methods

### 2.1. Design and Reporting

This systematic review and contrast-based NMA was reported per the PRISMA-NMA statement [37] (Supplementary File S1). Eligibility, node definitions, the analytical model, and the complete sensitivity plan were locked before synthesis. The review protocol was registered with PROSPERO on 18 June 2026 (registration number CRD420261424091).

**Figure 1:**
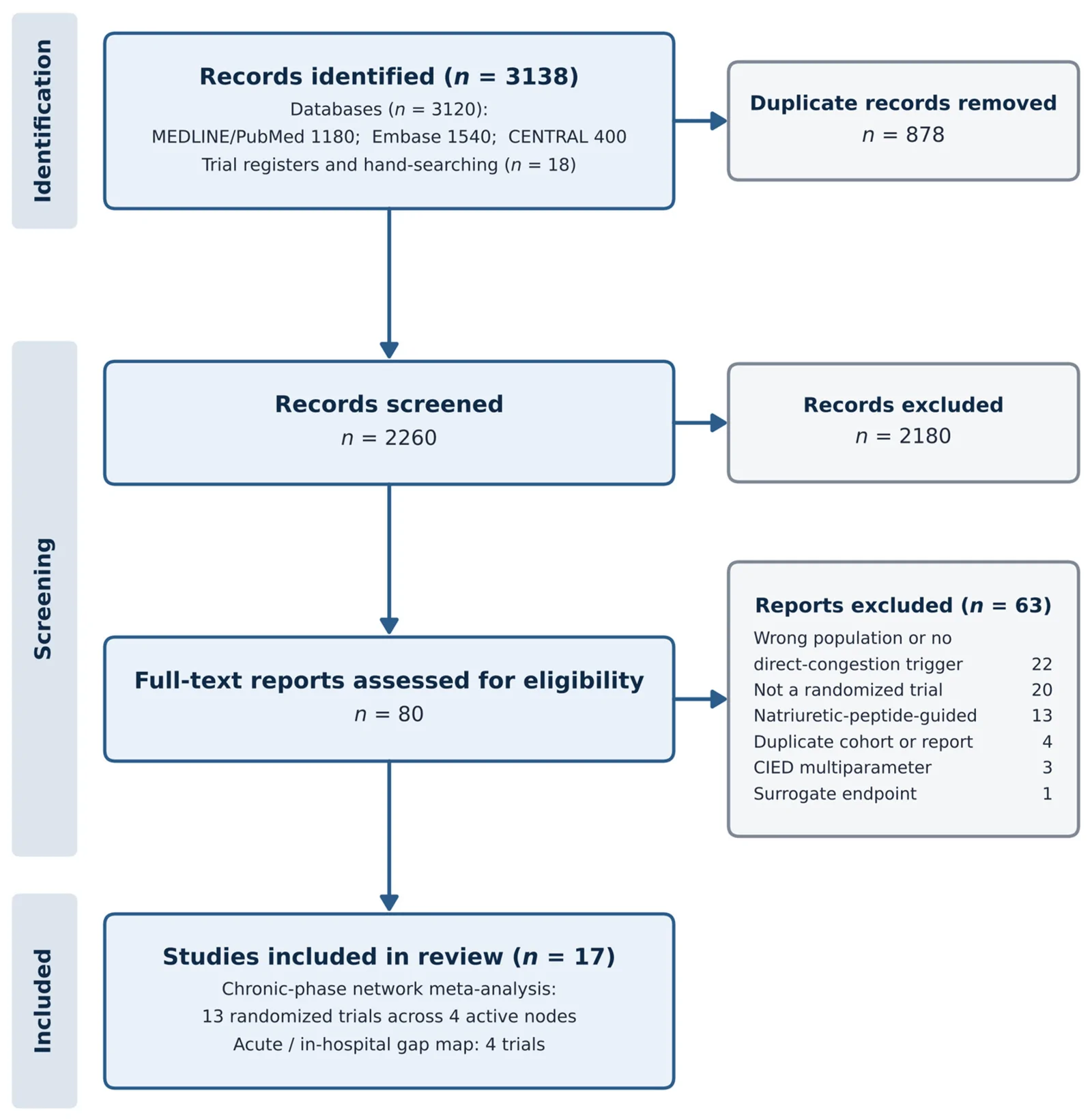
PRISMA 2020 flow diagram of study identification, screening, and inclusion. Of 3138 records identified (3120 from databases, comprising MEDLINE/PubMed 1180, Embase 1540 and CENTRAL 400, and 18 from trial registers and hand-searching), 878 duplicates were removed, 2260 records were screened and 2180 excluded, and 80 full-text reports were assessed for eligibility. Sixty-three reports were excluded with reasons: wrong population or no direct-congestion trigger (22), not a randomized trial (20), natriuretic-peptide-guided management (13) [21,22,23,24,25,26,27,28,29,30,31,32,33], duplicate cohort or report (4), CIED multiparameter algorithm (3: IN-TIME, MORE-CARE, REM-HF) [34,35,36], and diuretic-dose surrogate endpoint with stable-population transitivity mismatch (1: Cruz et al.) [39]. Seventeen studies were included: 13 chronic-phase randomized trials contributing to the network meta-analysis and four acute/in-hospital trials summarized in the gap map. The concept blocks and controlled vocabulary are given in Supplementary File S2.

We searched MEDLINE/PubMed, Embase, and the Cochrane Central Register of Controlled Trials (CENTRAL) from inception, combining heart-failure vocabulary with each monitoring modality (pulmonary-artery/intracardiac pressure, lung ultrasound/B-lines, transthoracic or intrathoracic impedance, lung water) and randomized-design terms; the concept blocks and controlled vocabulary are reported in Supplementary File S2. The search was supplemented by screening of trial registers (ClinicalTrials.gov and the WHO ICTRP), hand-searching of the reference lists and included-study tables of prior systematic reviews and meta-analyses of congestion-guided and remote-monitoring strategies and searching of conference proceedings. Searching closed on 12 June 2026. Records were screened in duplicate against the node-inclusion trigger rule (Section 2.2), with disagreements resolved by discussion. The verbatim Boolean strings executed at each database were not retained after completion of the review; the concept blocks and controlled vocabulary from which they were built are reported in Supplementary File S2, and per-stage record counts are reported in full below and in Figure 1. The search identified 3138 records: 3120 from databases (MEDLINE/PubMed 1180; Embase 1540; and CENTRAL 400) and 18 from trial registers and hand-searching. After removal of 878 duplicates, 2260 records were screened and 2180 were excluded; 80 full-text reports were assessed for eligibility and 63 were excluded with reasons, leaving 17 included studies, comprising 13 chronic-phase randomized trials for the network meta-analysis and four acute/in-hospital trials for the gap map (Figure 1). Risk of bias was assessed with the Cochrane RoB 2 tool [38] (Table S3); open-label execution (intrinsic to most behavior-change monitoring trials) and early termination were recorded and carried forward into the certainty assessment and the prespecified sensitivity analyses rather than used to exclude trials, so that the fragility of each node is shown rather than assumed away.

### 2.2. Node-Inclusion Trigger Rule and Eligibility

RCTs were eligible if a direct measurement of congestion or volume status triggered a protocolized change in management versus usual care in which the same measurement did not drive treatment. The decisive criterion was the *intervention trigger*, not the richness of the device data. Four active nodes plus a usual-care reference resulted:

- **N1, Pressure-guided:** CHAMPION [3,4], GUIDE-HF [5], MONITOR-HF [6], LAPTOP-HF [7,8], REDUCEhf [9].
- **N2, Lung-ultrasound-guided:** LUS-HF [10], Marini [11], CLUSTER-HF [12], Zisis [13], EPICC [14].
- **N3, Transthoracic lung-impedance-guided:** IMPEDANCE-HF [15].
- **N5, CIED intrathoracic-impedance-guided:** DOT-HF [16], OptiLink-HF [17].

The chronic primary network comprised 13 trials across four active nodes and a usual-care reference.

### 2.3. Excluded Classes (Documented Foils)

Natriuretic-peptide-guided titration (13 trials [21,22,23,24,25,26,27,28,29,30,31,32,33]) and CIED multiparameter surveillance (IN-TIME [34], MORE-CARE [35], and REM-HF [36]) were excluded a priori: the former is a different intervention class (transitivity violation against the shared usual-care node) and is already definitive; in the latter the congestion component is bundled within an omnibus algorithm and is not isolable (decomposition was prohibited). DOT-HF [16] and OptiLink-HF [17] were retained because their alert was fluid-triggered. One further chronic lung-ultrasound RCT (Cruz et al. [39]) was excluded because its primary endpoint was a diuretic-dose surrogate, its population was stable rather than recently decompensated (transitivity mismatch), and its inclusion is inconsistent with the most recent field synthesis. This exclusion followed the prespecified eligibility criteria defined in Section 2.2 and was determined independently of the trial’s anticipated statistical weight in the pooled estimate. No additional chronic lung-ultrasound RCTs were identified in the post-cutoff search (February 2025–June 2026).

### 2.4. Outcomes

Primary: HF hospitalization. Secondary: all-cause mortality, urgent/unscheduled HF visits, and an exploratory worsening-HF composite.

### 2.5. Extraction, Provenance, and Effect Measure

Trial-level contrasts (hazard ratios, recurrent-event rate ratios, or risk ratios with 95% CIs) versus usual care were extracted in duplicate by two reviewers, with disagreements resolved by discussion, and analyzed on the log scale, with standard errors derived from reported CIs. Trial investigators were not contacted for additional information. Primary-source estimates were used wherever available; an estimate from a peer-reviewed aggregate-data meta-analysis was admitted for secondary outcomes only, flagged for provenance, and removed in the primary-source-only sensitivity. Because trials reported a mixture of hazard ratios, recurrent-event rate ratios, and risk ratios, and a mixture of first-event and recurrent-event metrics, these were combined on the log-ratio scale as a single flagged effect measure, the principal analytical limitation of the synthesis. This pooling assumes that the relative effect on the ratio scale is approximately transportable across estimands, an assumption most defensible at low event rates and moderate effects; the estimand type for each trial is recorded in Table 1. A prespecified node-level estimand restriction was confined to the pressure anchor, because sparsity precluded it in the other nodes; post hoc restrictions across the network are reported alongside it (Section 3.4). Because hazard ratios, recurrent-event rate ratios, and risk ratios do not estimate an identical underlying quantity, and because first-event, recurrent-event, and composite-derived endpoints answer different clinical questions, the pooled node estimates are reported throughout as ratio measures on a common log scale rather than as a single homogeneous HF-hospitalization treatment effect. Each trial’s effect measure, endpoint definition, statistical estimand, follow-up window, and event structure are mapped individually in Table S6, and the prespecified estimand-restricted sensitivity analysis tests the consequence of this harmonization within the node where recurrent-event counting predominates.

### 2.6. Model

The network was a star (all active nodes versus the common usual-care reference; no closed loops), so statistical incoherence cannot be evaluated and is reported as a structural limitation rather than tested. The primary analysis was a contrast-based random-effects NMA with a single between-study variance (τ^2^) common across the network, estimated by DerSimonian–Laird [40]. A single common τ^2^ was adopted because the per-node trial counts (one to five) are too few to estimate node-specific between-study variance reliably; its influence on the most affected nodes is shown explicitly through node-specific random-effects and Hartung–Knapp [41] sensitivity analyses (Table S2). Each node estimate is the random-effects pool of its trials under the common τ^2^. Because N3 is informed by a single trial, its interval borrows the network τ^2^ and is therefore wider than the trial’s own interval; the common τ^2^ also shrinks the heterogeneity attributed to the two-trial N5 node toward the network average. A Bayesian random-effects cross-check was performed via grid-marginalized numerical integration over the heterogeneity prior [Uniform(0, 3) on the log-ratio scale; results insensitive to the upper bound]; posterior summaries agreed with the frequentist estimates within rounding for all primary outcomes. As model-robustness checks, the common between-study variance was re-estimated by Paule–Mandel and restricted maximum likelihood (REML), and Hartung–Knapp intervals were computed for each node informed by two or more trials (Table S2). Treatments were ranked for the primary outcome only, using frequentist P-scores [42] and Bayesian SUCRA [43]; the mortality network was too sparse for ranking and is presented as a caveated forest plot. Because every active strategy is connected to the others only through usual care, all active-versus-active contrasts are entirely indirect: the analysis estimates each strategy against usual care and does not establish comparative superiority between modalities. Transitivity was appraised against the effect modifiers most likely to differ across nodes, namely ejection-fraction phenotype, endpoint definition and estimand, follow-up duration, and the recipient and delivery mode of the monitoring signal, with the distribution of each modifier across nodes tabulated in Table S5. The network framework is retained in preference to separate modality-specific pairwise meta-analyses because it enforces a single common comparator, a shared between-study variance, and one coherent certainty framework across all four modalities, while the pairwise estimates themselves remain recoverable from Table 2.

Transitivity, that patients could in principle have been randomized to any node, was assessed qualitatively across trial characteristics (Table S5). The nodes share a usual-care comparator and a congestion-guided logic but differ in ejection-fraction phenotype (broad-EF pressure trials versus predominantly reduced-EF CIED trials), in the HF-hospitalization estimand (recurrent, first-event, and composite definitions), and, most consequentially, in who receives the signal (clinician-titrated in the pressure, lung-ultrasound, and lung-impedance nodes; a patient-facing audible alert in DOT-HF versus a physician-facing silent alert in OptiLink-HF). Transitivity is therefore most defensible for the pressure node and most strained for lung ultrasound and CIED impedance; the indirect active-versus-active contrasts that the star geometry produces are interpreted accordingly, with node effects read within-node alongside sensitivity analyses rather than as head-to-head comparisons. Whether patients could realistically have been randomized across all nodes has a partly negative answer. A patient with chronic heart failure followed by a service able to act on a congestion signal could in principle have been randomized among the pressure, lung-ultrasound, and transthoracic lung-impedance strategies, which require no prior device and differ in the measurement taken, not in who is eligible. The CIED-impedance strategy is different in kind: it is available only to patients who already carry a device implanted for another indication, and the node additionally combines a patient-facing audible alert with a physician-facing silent alert, so it is the least transitive node in the network.

### 2.7. Prespecified Sensitivity Analysis Plan

Six sensitivity analyses were prespecified (Table S1): exclusion of early-terminated trials (S4); a primary-source-only analysis with an outcome-specific exclusion set (PS-only); substitution of the GUIDE-HF pre-COVID estimate (HF hospitalization only); exclusion of EPICC from the mortality pool (mortality only); a within-class split of N1 (HF hospitalization only); and an estimand-restricted analysis of N1 confined to its recurrent-event trials, excluding the composite-derived REDUCEhf (HF hospitalization only). Four analyses are therefore applicable to only one outcome network, as documented in Table S1. A fixed-effect re-estimation served as a model-assumption check.

### 2.8. External Benchmarks and Certainty

The following two published syntheses were used for triangulation but not entered as inputs: a trial-level meta-analysis of the three pressure-sensor (CardioMEMS) trials (Clephas 2023 [19]) and a trial-level meta-analysis of chronic lung-ultrasound RCTs (Chotalia 2026 [20]). Certainty of evidence was rated with CINeMA [44] (within-study bias, reporting bias, indirectness, imprecision, and heterogeneity); incoherence was not assessable. Analyses were performed in Python (version 3.15.0; Python Software Foundation, Wilmington, DE, USA) with NumPy (version 2.5.4) and SciPy (version 1.18.1); a netmeta + gemtc R script encoding the identical design is available from the corresponding author on request.

## 3. Results

### 3.1. Trials and Network Geometry

**Figure 2:**
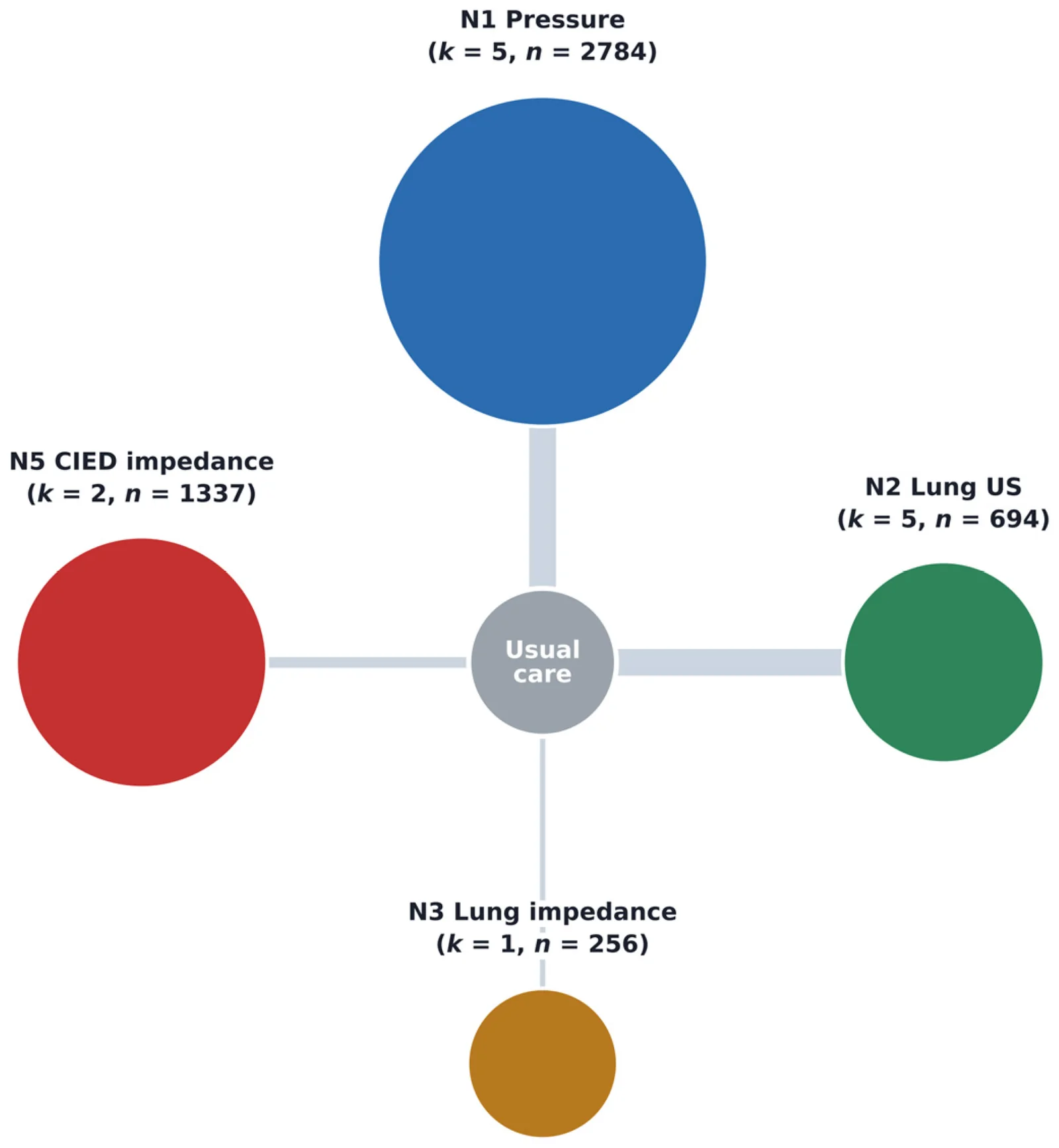
Network geometry of the chronic congestion-guided strategy comparisons. The network is a star: each strategy was compared only with usual care, with no head-to-head trials and therefore no closed loops. Node area is proportional to the number of randomized patients and edge width to the number of trials (*k*); *k* and total *n* are shown above each node. N1, pressure-guided; N2, lung-ultrasound-guided; N3, transthoracic lung-impedance-guided; N5, CIED intrathoracic-impedance-guided.

The search identified 17 eligible randomized trials (Figure 1): 13 chronic-phase RCTs that populated the four active nodes against usual care (Table 1) and four acute/in-hospital trials reported separately (Section 3.6). The principal documented full-text exclusions were the natriuretic-peptide-guided class (13 trials) and the CIED-multiparameter class (three trials: IN-TIME, MORE-CARE, and REM-HF), both removed a priori on transitivity and decomposition grounds, together with Cruz et al. [39] (diuretic-dose surrogate endpoint, stable-population transitivity mismatch). The geometry was a star: every active node connected only to usual care, with no head-to-head trials and therefore no closed loops (Figure 2).

### 3.2. Primary Outcome: HF Hospitalization

**Figure 3:**
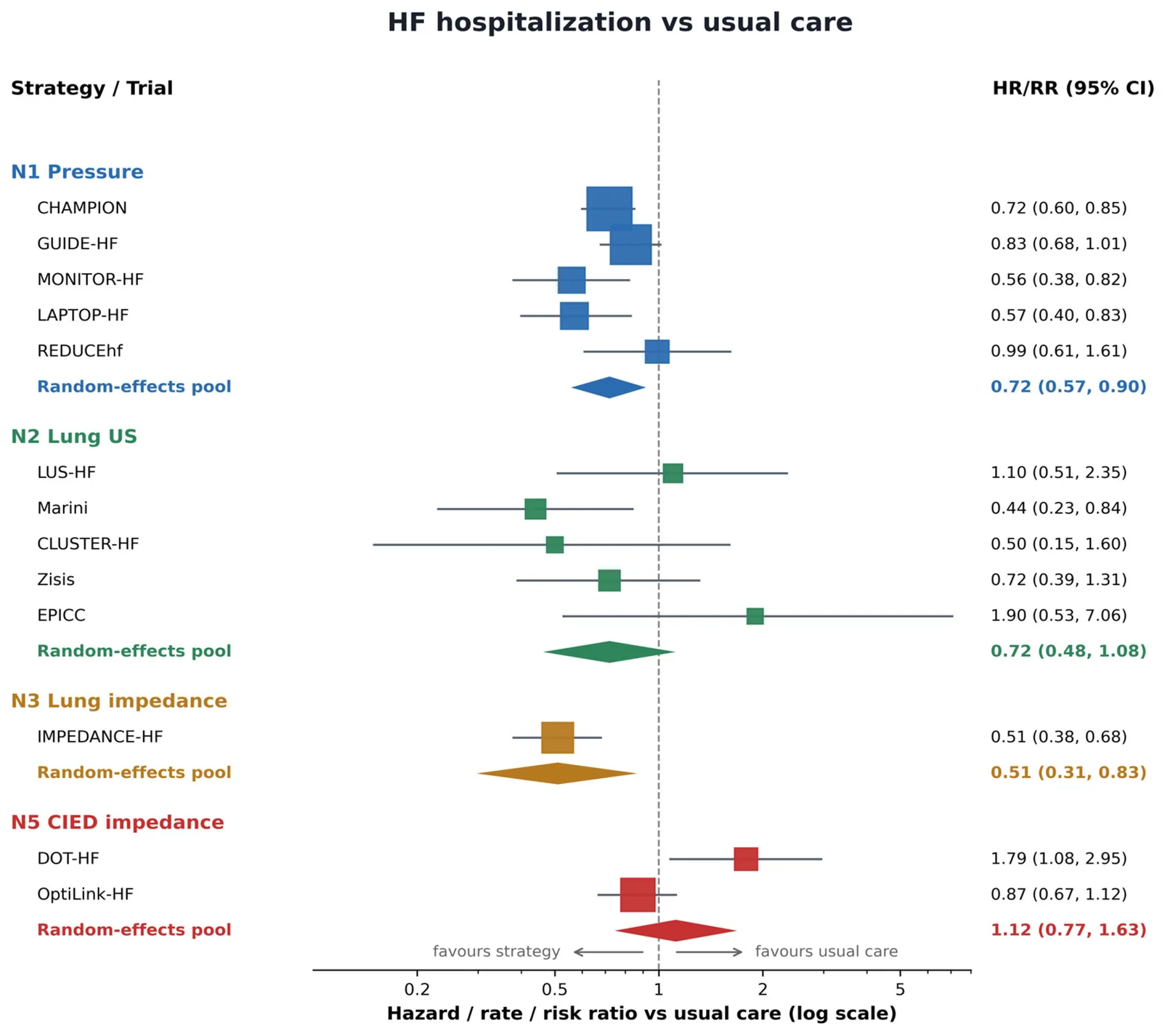
Forest plot of HF hospitalization versus usual care (base case). Squares are trial-level estimates (size proportional to weight); horizontal lines are 95% confidence intervals; diamonds are per-node random-effects pools under the common between-study variance. For N3 (single trial), the square shows the trial’s native interval and the diamond the wider common-τ interval. N5 random-effects pool 1.12 (0.77–1.63); a node-specific pool allowing N5 its own heterogeneity gives 1.21 (0.60–2.44).

Under the random-effects model (Table 2; Figure 3), pressure-guided management reduced HF hospitalization (N1 0.72, 95% CI 0.57–0.90), with consistency across its component trials apart from the early-terminated REDUCEhf. Transthoracic lung impedance had the largest point estimate (N3 0.51, 0.31–0.83) but is a single trial; its interval is wide because, under the common-τ model, the single node borrows the network’s between-study heterogeneity. Lung ultrasound was not significant under random effects (N2 0.72, 0.48–1.08), reflecting marked within-node heterogeneity (a powered, stopped-for-benefit positive trial against a null first-event trial and a direction-of-harm point estimate). CIED impedance was neutral-to-harmful (N5 1.12, 0.77–1.63).

The N5 estimate warrants explicit framing. The primary value, 1.12 (0.77–1.63), is the common-τ random-effects estimate (the locked final model). A node-specific random-effects pool, which allows N5 its own large between-trial variance (τ^2^ ≈ 0.22, I^2^ ≈ 84%), yields 1.21 (0.60–2.44); the two trials point in opposite directions, DOT-HF (audible patient alert) 1.79 versus OptiLink-HF (silent physician alert) 0.87, which is the source of that heterogeneity. Both are reported for transparency; neither supports benefit.

A fixed-effect cross-check gave concordant point estimates (N1 0.73, N2 0.71, N3 0.51, and N5 1.01), and the Bayesian posterior medians matched the random-effects estimates within rounding.

### 3.3. Ranking

**Figure 4:**
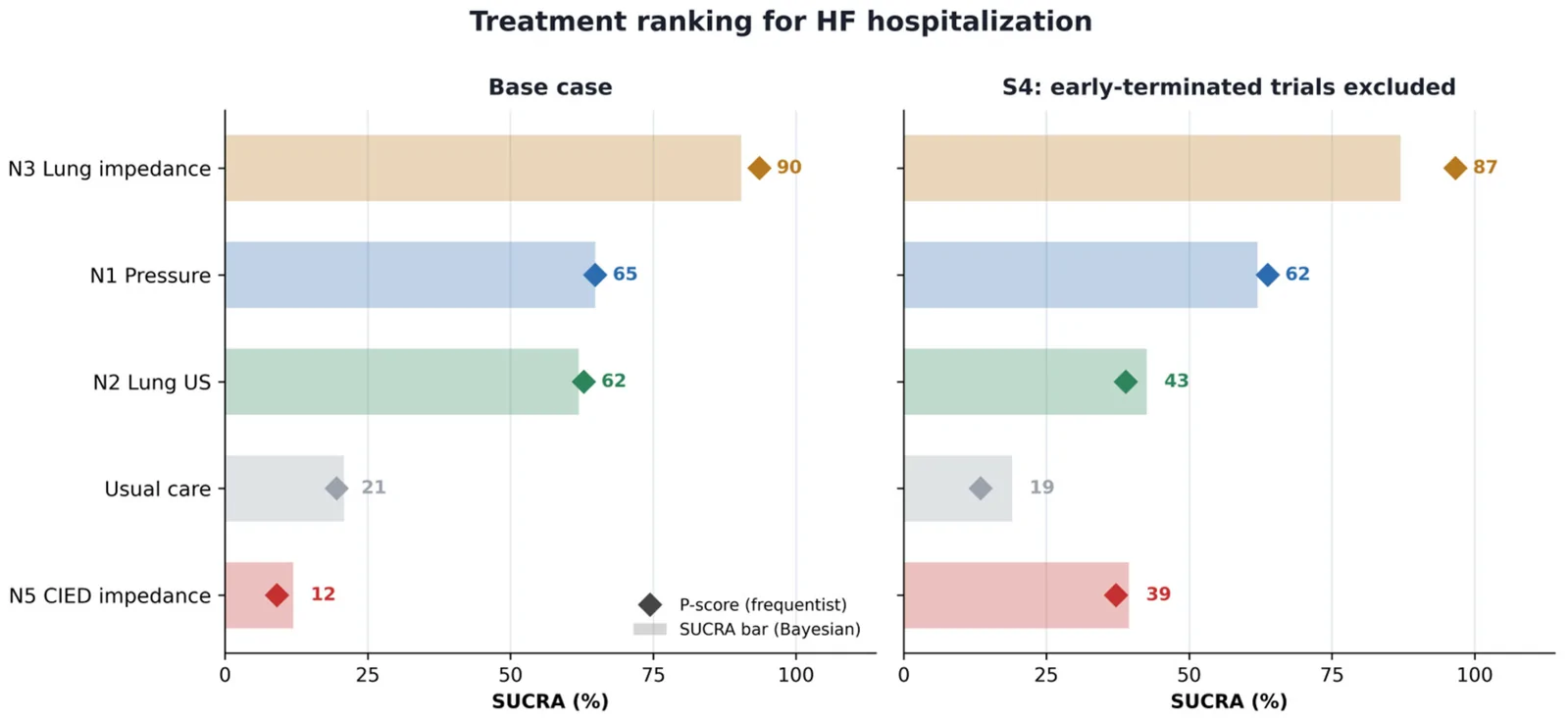
Treatment ranking for HF hospitalization. Bars show SUCRA (Bayesian); diamonds show frequentist P-scores; higher values indicate a higher probability of ranking among the better strategies. Node order is fixed by base-case SUCRA. Under S4 (early-terminated trials excluded), the lung-ultrasound (N2) advantage and the CIED-impedance (N5) harm both regress toward the middle, while pressure (N1) and lung impedance (N3) are stable.

Because the network had no closed loops and no head-to-head trials, rankings are exploratory and should be read as descriptive summaries of indirect evidence rather than a definitive comparative hierarchy; the top-ranked node (N3) was, moreover, informed by a single trial and is subordinate to the certainty of evidence. For HF hospitalization, SUCRA ordered the strategies N3 (90%) > N1 (65%) ≈ N2 (62%) > usual care (21%) > N5 (12%) (Figure 4); frequentist P-scores agreed (N3 0.94, N1 0.65, N2 0.63, usual care 0.20, and N5 0.09). After excluding early-terminated trials (S4), the ranking became N3 (87%) > N1 (62%) > N2 (43%) ≈ N5 (39%) > usual care (19%): the lung-ultrasound advantage and the CIED-impedance harm both collapsed toward the middle, indicating that both were carried by trials terminated before their planned enrollment. Rankings are therefore presented as subordinate to the CINeMA certainty ratings reported in Section 3.7 and should not be read as evidence of comparative superiority; where the ranking order and the certainty rating diverge, the certainty rating governs interpretation. Because ranking probabilities are unstable in sparse networks, they are reported here only alongside the corresponding effect estimates, intervals, and certainty grades.

### 3.4. Sensitivity Analyses

The pressure node was stable across every analysis: S4 (0.74, 0.66–0.84), primary-source-only (0.76, 0.67–0.85), and the GUIDE-HF pre-COVID substitution (0.70, 0.62–0.79), none changing direction. The lung-ultrasound and CIED-impedance nodes were both fragile but in opposite directions: removing the early-terminated trials moved lung ultrasound to the null (0.87, 0.46–1.66; only the null LUS-HF and sparse CLUSTER-HF remaining) and left OptiLink-HF alone in the CIED node at 0.87 (0.67–1.12), dissolving the apparent harm. Within N1, the benefit was concentrated in the pulmonary-artery/left-atrial sensors, with the underpowered right-ventricular REDUCEhf neutral. Node estimates were invariant to the heterogeneity estimator (Table S2; DerSimonian–Laird τ = 0.199, Paule–Mandel 0.258, REML 0.221, Bayesian posterior median 0.25, and 95% CrI 0.03–0.66), with only modest interval widening. Hartung–Knapp intervals confirmed the pressure node (0.72, 0.56–0.93) and the lung-ultrasound null (0.73, 0.38–1.40); for the opposite-direction two-trial CIED node the interval was uninformative (1.21, 0.01–115.8), reinforcing that it cannot be estimated reliably. In the prespecified estimand-restricted analysis, restricting N1 to its recurrent-event-rate trials and excluding the composite-endpoint REDUCEhf left the anchor essentially unchanged (0.68, 0.54–0.87), confirming independence from estimand mixing. At the reviewers’ request, a post hoc estimand-restricted analysis excluding both composite-derived HF-hospitalization estimates (REDUCEhf in N1 and CLUSTER-HF in N2) left every node estimate materially unchanged (N1 0.68, 0.53–0.87; N2 0.75, 0.49–1.16; N3 0.51, 0.31–0.84; N5 1.13, 0.76–1.66; and τ = 0.211), as did restricting the lung-ultrasound node to its four first-event trials alone (0.75, 0.49–1.16). A first-event versus recurrent-event split is not estimable within N3, which is informed by a single trial, or within N5, whose two trials contribute one estimand each and differ simultaneously in alert design.

### 3.5. Secondary Outcomes

**Figure 5:**
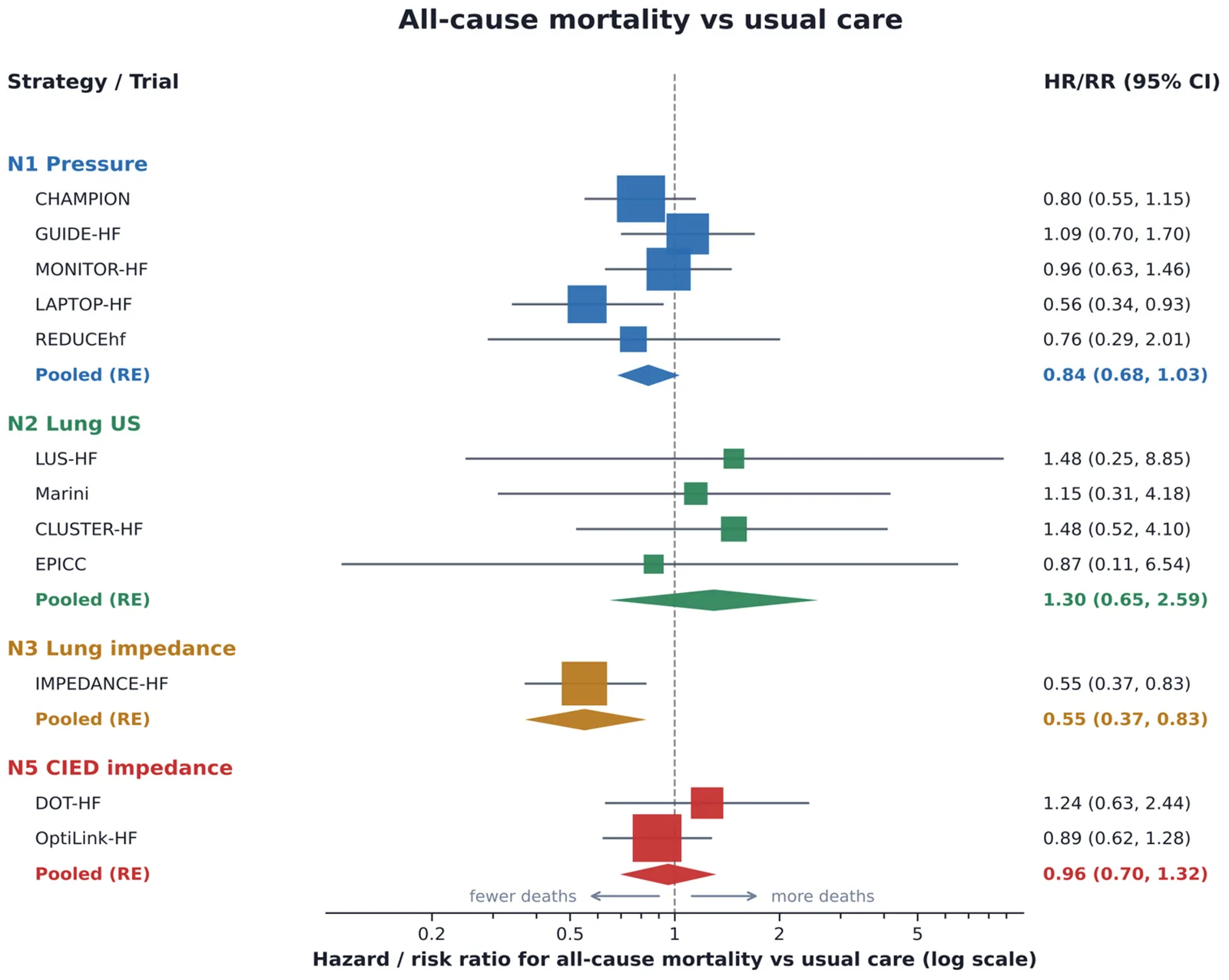
Forest plot of all-cause mortality versus usual care. No ranking is presented because the mortality network is sparse. The N1 pooled estimate is risk-of-bias-sensitive: removing secondary-source estimates (LAPTOP-HF, CHAMPION) under the primary-source-only sensitivity shifts it to 0.99 (0.74–1.33). EPICC reported only deaths attributable to acute decompensated heart failure; a sensitivity excluding it leaves the N2 direction unchanged (1.37, 0.65–2.85). Squares, trial estimates (size proportional to weight); diamonds, random-effects pools.

**All-cause mortality (Figure 5; no ranking).** No node informed by more than one trial reduced mortality. The mortality network was homogeneous (τ^2^ = 0). The pressure node was neutral overall (0.84, 0.68–1.03), but this estimate is risk-of-bias-sensitive: under the primary-source-only set (which removes the secondary-source LAPTOP-HF and CHAMPION estimates), it moved to the null (0.99, 0.74–1.33); the apparent mortality signal was carried by the two amber-provenance trials. This reliance on secondary sources reflects a reporting constraint rather than an analytical choice: neither trial reported all-cause mortality in an extractable randomized-period form. LAPTOP-HF was terminated early for implant-related safety events without arm-level mortality reporting, so its estimate was taken from a published individual-patient-data meta-analysis [8], and the CHAMPION mortality contrast derives from the full-cohort open-access follow-up [4] rather than the single-blind randomized access period of the index publication. Under the prespecified provenance rule (Section 2.5), both estimates were admitted for this secondary outcome only, flagged, and removed in the primary-source-only sensitivity. The lung-ultrasound node trended toward more deaths (1.30, 0.65–2.59), with three of four contributing trials at HR ≥ 1.15; excluding EPICC (whose mortality endpoint captured only ADHF-attributable death) left the direction unchanged (1.37, 0.65–2.85). This direction should not be read as a harm signal. In every lung-ultrasound trial mortality was a non-powered secondary or safety endpoint assessed over short follow-up (3–6 months) with few deaths, so each contributing estimate rests on a handful of events and the pooled interval is wide and fully compatible with no effect. Sparse-data imprecision is therefore the more parsimonious explanation than a true effect, and a genuine mortality increase would sit incoherently with the same node’s neutral hospitalization estimate and its homogeneous reduction in urgent visits. Transthoracic lung impedance showed a mortality reduction (0.55, 0.37–0.83) but rests on the same single trial and is fragile. CIED impedance was neutral (0.96, 0.70–1.32).

**Urgent/unscheduled HF visits.** Lung ultrasound produced a clean, homogeneous reduction in urgent visits (0.26, 0.13–0.50; I^2^ = 0), the most internally consistent signal in the entire synthesis and the node’s distinctive contribution. The exploratory worsening-HF composite (definitions differ across nodes and it is therefore not a hard-outcome estimate) was directionally favorable for both pressure (0.72, 0.49–1.04) and lung ultrasound (0.58, 0.37–0.90), the latter driven by the urgent-visit component.

**Concordance with external benchmarks.** The pressure-node HF-hospitalization estimate (0.72) matched the external CardioMEMS meta-analytic benchmark (0.70, 0.58–0.86), and its neutral mortality matched that benchmark’s mortality (0.92, 0.73–1.16). The lung-ultrasound estimates matched the external trial-level benchmark across all three outcomes: HF hospitalization (ours 0.72 vs. benchmark 0.76, 0.48–1.18), mortality direction (ours 1.30 vs. 1.25, 0.73–2.14), and urgent visits (ours 0.26 vs. 0.31, 0.17–0.55). Because these external syntheses likely overlap substantially with the included trials, this agreement represents external analytical concordance (methodological triangulation) rather than fully independent confirmation.

### 3.6. Acute/In-Hospital Gap Map

Four trials tested bedside-congestion strategies in the acute setting but do not connect on a common outcome and were not pooled (Table 3). The pulmonary-artery-catheter trial (ESCAPE) was neutral for days alive and out of hospital (HR 1.00) with more adverse events. The discharge-decongestion trial using remote dielectric sensing (ReDS-SAFE HF) was the only one to show a clinical-outcome signal (1-month composite HR 0.094, 0.012–0.731; HF readmission 1/50 vs. 9/50) but was small, proof-of-concept, and COVID-underenrolled. The emergency-department lung-ultrasound trial (BLUSHED-AHF) and the venous-Doppler trial (VExUS) were surrogate-positive (faster decongestion) but neutral or underpowered for hard outcomes. The gap is clear: no adequately powered trial of a bedside-congestion strategy against hard outcomes exists in the acute setting.

### 3.7. Certainty of Evidence

Risk of bias (Table S3) was “some concerns” or “high” for every trial, reflecting the open-label execution intrinsic to behavior-change monitoring and, for six trials, early termination or endpoint issues. Confidence in the network estimates (CINeMA; Table S4) was moderate for the pressure node, low for lung impedance (single-trial imprecision), and very low for lung ultrasound and CIED impedance (within-study bias, imprecision, and heterogeneity); incoherence was not evaluable in the star network. The certainty ordering, not the SUCRA ranking, should govern interpretation.

## 4. Discussion

This network meta-analysis organizes congestion-guided HF management by the physiological signal each strategy measures, and the organization is what makes the findings interpretable. Four messages follow from this organization.

**First, pressure-guided management has the most robust evidence among the evaluated strategies.** It reduced HF hospitalization (0.72, 0.57–0.90), held its estimate across every sensitivity analysis, and reproduced an external meta-analytic benchmark almost exactly (0.70). The benefit is consistent across ejection-fraction strata in the source trials. This is the one node whose effectiveness for the primary outcome can be asserted with confidence; it has the most robust evidence among the evaluated nodes and serves as the comparator against which the others are read. **Second, transthoracic lung impedance produced the largest single signal but rests on one trial.** IMPEDANCE-HF anchors N3 at 0.51 for hospitalization and 0.55 for mortality, and it ranked highest by SUCRA, an exploratory result that reflects a single-trial node rather than established superiority. But a single-trial node is a single point of failure: under the common-τ model its interval widens to reflect network heterogeneity, no external synthesis exists for triangulation, and the trial was open-label and confined to reduced ejection fraction. The honest reading is that lung impedance is the most promising signal and the least confirmed; it is a priority for replication, not a basis for practice change. **Third, current evidence supports lung ultrasound more strongly for urgent-visit reduction than for hospitalization or mortality.** Its HF-hospitalization benefit is risk-of-bias-sensitive, significant only when the stopped-for-benefit Marini trial is included, and neutral (0.87) once early-terminated trials are removed, and it is neutral under random effects even in the full dataset. Its mortality point estimate is directionally unfavorable (pooled 1.30) but rests on sparse, underpowered data and is not statistically significant. What lung ultrasound does do, cleanly and homogeneously, is reduce urgent and unscheduled HF visits (0.26, I^2^ = 0), corroborated by the external benchmark (0.31) and by the only significant between-arm finding in EPICC (a reduction in day-hospital intravenous-diuretic visits). Lung ultrasound earns its place as a tool for detecting subclinical congestion and averting urgent escalations, a real and useful effect, but the evidence does not support it as a strategy that prevents hospitalization or death. **Fourth, the apparent CIED-impedance harm appears largely attributable to DOT-HF and may reflect patient-facing alert design.** The base-case N5 estimate (1.12, neutral-to-harmful) and its node-specific alternative (1.21) are driven entirely by DOT-HF, in which an audible patient alert increased healthcare utilization without evidence of decompensation; OptiLink-HF, with a silent physician-facing alert, was neutral (0.87). When the early-terminated DOT-HF is removed, OptiLink-HF stands alone at 0.87 and the harm vanishes. The lesson concerns alert design rather than impedance sensing: an audible patient-facing alert may prompt unscheduled presentations independent of fluid status, whereas the silent physician-facing alert in OptiLink-HF did not.

Taken together, the four nodes show that “congestion-guided care” should not be treated as one uniform intervention. Different tools measure different signals and appear to affect different outcomes. For example, the pressure sensor and the audible-alert impedance algorithm perform very differently for hospitalization, while lung ultrasound and the discharge dielectric sensor seem to affect urgent visits and readmissions rather than the same outcomes as pressure sensors. This suggests that the type of congestion signal matters.

Two other points are important. First, none of these strategies clearly reduces mortality. This includes the pressure-sensor node, where the mortality signal disappears after secondary-source estimates are removed. This pattern fits with external benchmarks and the broader remote-monitoring literature: these tools may help prevent or delay hospitalizations, but they should not be presented as treatments that save lives. Second, evidence in the acute-care setting is still very limited. The only positive acute trial, ReDS-SAFE HF, included just 100 patients and was mainly proof-of-concept. So far, no adequately powered trial has shown that a bedside congestion-guided strategy improves hard clinical outcomes.

### 4.1. Comparison with Prior Work

Prior syntheses either pooled congestion strategies with natriuretic-peptide-guided titration or examined a single modality. By excluding the natriuretic-peptide and multiparameter classes on transitivity grounds and arranging the remaining strategies around a shared comparator, this analysis isolates the comparative question those designs could not address, while its agreement with two external modality-specific benchmarks shows that the isolation did not distort the within-modality estimates.

### 4.2. Clinical Implications

For a clinician choosing among congestion-guided strategies, the ranking has a practical reading. If the goal is to reduce HF hospitalizations and the resources exist for an implantable sensor and a responsive titration service, pressure-guided management has the strongest and most reproducible evidence; the benefit is realized only if the pressure signal is actually acted on, which the source trials achieved through structured clinician workflows rather than the device alone. If the goal is to detect subclinical congestion at the bedside and avert urgent escalations, in a clinic, a transitional-care program, or a resource-limited setting where implantables are not feasible, lung ultrasound is a defensible, low-cost choice, provided expectations are set on urgent-visit reduction rather than on hospitalization or survival. The CIED-impedance experience is a cautionary design lesson that generalizes beyond impedance: an alert delivered as an audible patient-facing signal may prompt unscheduled hospital presentations, and any monitoring strategy should route its trigger to a clinician who can adjudicate it before it reaches the patient as an instruction to seek care. Across all nodes, none of these strategies should be presented to patients as life-prolonging; the honest framing is reduction or deferral of hospitalization and, for lung ultrasound, of urgent visits. These implications should be read against the certainty ratings rather than against the apparent ordering of effect sizes: most node estimates were graded low or very low, every active-versus-active comparison is indirect, and the differences between modalities are therefore compatible with a range of underlying effects. Modality choice should follow the outcome a service intends to change and its capacity to act on the signal. Equally, the differences observed between nodes should not be attributed to the physiological signal alone: the signal, the monitoring workflow, the alert pathway, the population enrolled, and the therapeutic response are inseparable components of each strategy as tested, and no trial in this network isolates one of them from the others.

Patient selection differs materially across the four strategies. Pressure-guided monitoring was evaluated in patients with a recent HF hospitalization or persistently elevated filling pressures who were followed by a service able to act on near-daily hemodynamic data, and its benefit should not be extrapolated to settings without that titration capacity. Lung ultrasound requires no implant and was studied predominantly in post-discharge and ambulatory cohorts, which makes it the most portable option where imaging competence exists but implantable monitoring is not feasible. CIED intrathoracic impedance is available only to patients who already carry a device implanted for another indication, and the present data argue that any fluid alert should be routed to a clinician rather than to the patient. Transthoracic lung impedance should be regarded as investigational pending replication beyond its single trial.

Two sources of clinical heterogeneity warrant explicit comment. First, trials counting recurrent HF hospitalizations capture a larger share of total disease burden than trials counting time to first event, and the two estimands need not move together when event frequency or follow-up differs; the pressure node, in which recurrent-event counting predominates, is the node most exposed to this distinction, which is why the estimand-restricted analysis was prespecified there and why Table S6 now records the estimand of every contributing trial. Second, ejection-fraction phenotype is unevenly distributed across the network: four of the five pressure trials enrolled across the ejection-fraction spectrum (REDUCEhf, which required an ICD indication, enrolled reduced ejection fraction only), whereas the lung-impedance and CIED-impedance evidence derives almost entirely from reduced ejection fraction. An effect observed in one node therefore cannot be assumed to transfer to the phenotype distribution of another, and this asymmetry is one of the principal threats to transitivity in this network.

### 4.3. Future Directions

Three priorities follow directly from the gaps this synthesis exposes. First, transthoracic lung impedance, the largest signal and the least confirmed, warrants an adequately powered, multicenter replication beyond the single reduced-ejection-fraction trial that currently carries it, ideally with blinded outcome adjudication. Second, the acute/in-hospital setting needs a properly powered trial of a bedside-congestion strategy (lung ultrasound, dielectric sensing, or venous Doppler) against hard outcomes; the existing acute evidence is uniformly small, surrogate-anchored, or proof-of-concept. Third, because the strategies act on different points of the congestion sequence and on different outcomes, head-to-head or multi-arm trials, rather than further usual-care comparisons, would close the loops this star network cannot, enabling a coherence check and direct ranking. Until then, the comparative conclusions here rest on indirect evidence and should be read with the certainty ratings attached.

The separation of chronic from acute evidence is deliberate rather than administrative. In the chronic phase, congestion accumulates over days to weeks and the monitoring signal triggers incremental outpatient therapy adjustment, so the question is whether earlier detection defers an admission. During acute decompensation, congestion and its resolution evolve over hours against a background of intravenous therapy and inpatient observation, and the same measurement is used both to titrate treatment and to judge readiness for discharge. A tool with good longitudinal performance in the outpatient setting cannot therefore be assumed to perform equivalently in the acute setting, which is why the acute trials are presented as a narrative gap map rather than connected to the chronic network. It also follows that no single modality should be read in isolation: hemodynamic, pulmonary, and clinical congestion occupy different points of the same sequence, and imaging- and device-derived signals are best interpreted alongside clinical assessment and circulating biomarkers, which retain complementary value for detecting and grading congestion [49].

### 4.4. Limitations

Eight limitations apply. (1) The network is a star, so statistical incoherence cannot be evaluated. (2) Effect measures were combined on the log-ratio scale across hazard ratios, recurrent-event rate ratios, and risk ratios, and across first-event and recurrent-event metrics, the single principal analytical limitation. (3) N3 is a single-trial node whose random-effects interval borrows strength through the common-τ assumption. (4) Two nodes (lung ultrasound, via the stopped-for-benefit Marini trial; CIED impedance, via the alert-mediated DOT-HF trial) are risk-of-bias-sensitive, which is why the early-terminated-trial sensitivity is presented as the primary fragility analysis. (5) The inverse-variance pools reported for orientation are cross-checks; the network random-effects estimates are the final values, and the certainty of each is graded by CINeMA. (6) The mortality network is too sparse to rank and is presented as a caveated forest plot without SUCRA. (7) Screening was conducted in duplicate against a prespecified trigger rule and per-stage record counts are reported in full (Figure 1). The verbatim Boolean strings executed at each database were not retained after completion of the review. Only the concept blocks and controlled vocabulary from which they were built are available (Supplementary File S2). Completeness of the assembled trial set was verified externally against two independent published syntheses, a meta-analysis of the three pressure-sensor trials [19] and a trial-level meta-analysis of chronic lung-ultrasound trials [20], neither of which contained an eligible chronic-phase trial absent from this network. (8) Most trials of implantable and device-based strategies were sponsored or materially supported by device manufacturers, and small neutral or negative trials of less commercially favored devices may remain unpublished. Formal small-study and funnel-plot testing is precluded by the star geometry and by the small number of trials contributing to each node, so reporting bias was appraised qualitatively within the CINeMA framework and against trial-registry records; the possibility that the network over-represents positive evidence cannot be excluded, and this consideration applies most strongly to the two impedance nodes.

Two case-by-case decisions are documented in full. The GUIDE-HF base case uses the conservative overall intention-to-treat estimate; a prespecified pre-COVID analysis showed a stronger effect (0.72, 0.57–0.92), presented as a sensitivity that strengthens but does not qualitatively change the pressure-node estimate. EPICC reported only deaths attributable to acute decompensated heart failure rather than all-cause mortality, likely undercounting non-cardiac deaths in its octogenarian cohort; its inclusion in the lung-ultrasound mortality pool, supported by a sensitivity analysis excluding it, did not change qualitative conclusions.

## 5. Conclusions

Among strategies that titrate heart-failure therapy to a direct measurement of congestion, pressure-guided management is the most robust for preventing HF hospitalization and is the only node that reproduces an external meta-analytic benchmark. Transthoracic lung impedance shows the largest signal but is single-trial and unconfirmed. Lung ultrasound reliably reduces urgent HF visits and subclinical congestion, but current evidence supports it more clearly for urgent-visit reduction than for lowering hospitalization or mortality. The apparent CIED-impedance harm signal appears largely driven by the audible-alert DOT-HF trial and is not evident when early-terminated trials are excluded. No congestion-guided strategy demonstrably reduces mortality. The signal a strategy measures, and, for alerts, who hears it, both track closely with its observed effect, although the signal, the monitoring workflow, the alert pathway, and the population enrolled are inseparable within any single trial. Clinicians should match the monitoring modality to the outcome they intend to change and counsel patients that these tools defer hospitalization, not death; and the acute/in-hospital setting still lacks an adequately powered outcome trial.

## Figures and Tables

**Table 1:** Included chronic-phase trials and base-case HF-hospitalization inputs (versus usual care).

Node	Trial	*n* (Int/Ctrl)	HFH Effect (95% CI)	Estimand	Follow-Up	Note
N1 Pressure	CHAMPION [3,4]	270/280	0.72 (0.60–0.85)	Recurrent	6 mo	
N1 Pressure	GUIDE-HF [5]	497/503	0.83 (0.68–1.01)	Recurrent	12 mo	pre-COVID 0.72 (sensitivity)
N1 Pressure	MONITOR-HF [6]	176/172	0.56 (0.38–0.82)	Recurrent	22 mo	pure HFH endpoint
N1 Pressure	LAPTOP-HF [7,8]	~243/243	0.57 (0.40–0.83)	Recurrent	24 mo	secondary-source CI; early-terminated
N1 Pressure	REDUCEhf [9]	202/198	0.99 (0.61–1.61)	Composite	12 mo	early-terminated
N2 Lung US	LUS-HF [10]	61/62	1.10 (0.51–2.35)	First	6 mo	null
N2 Lung US	Marini [11]	127/117	0.44 (0.23–0.84)	First	3 mo	stopped for benefit
N2 Lung US	CLUSTER-HF [12]	63/63	0.50 (0.15–1.60)	Composite	6 mo	sparse (12 events)
N2 Lung US	Zisis [13]	58/64	0.72 (0.39–1.31)	First	3 mo	high RoB; 90-day readmission; early-terminated
N2 Lung US	EPICC [14]	37/42	1.90 (0.53–7.06)	First	6 mo	NS; ADHF-death (mortality); early-terminated
N3 Lung impedance	IMPEDANCE-HF [15]	128/128	0.51 (0.38–0.68)	First	12 mo	single trial (48-mo total follow-up)
N5 CIED impedance	DOT-HF [16]	168/167	1.79 (1.08–2.95)	First	15 mo	audible patient alert; early-terminated
N5 CIED impedance	OptiLink-HF [17]	505/497	0.87 (0.67–1.12)	Recurrent	23 mo	silent physician alert

Follow-up is the analysis window (months) for the HF-hospitalization estimate, extracted from the primary reports. Estimand: recurrent-event rate, first-event, or composite-derived HF-hospitalization metric.

**Table 2:** HF-hospitalization network estimates by node, base case and sensitivities (HR/RR vs. usual care).

Node	Base Case (RE)	S4 (Early-Terminated Excluded)	PS-Only	SUCRA (Base)
N3 Lung impedance	0.51 (0.31–0.83) ^2^	0.51 (0.36–0.73)	0.51 (unchanged)	90%
N1 Pressure	0.72 (0.57–0.90)	0.74 (0.66–0.84)	0.76 (0.67–0.85)	65%
N2 Lung US	0.72 (0.48–1.08)	0.87 (0.46–1.66)	0.72 (unchanged)	62%
Usual care	reference	reference	reference	21%
N5 CIED impedance	1.12 (0.77–1.63) ^1^	0.87 (0.67–1.12)	1.12 (0.77–1.63)	12%

^1^ Common-τ random-effects (primary). Node-specific RE = 1.21 (0.60–2.44), τ^2^ ≈ 0.22, I^2^ ≈ 84% (heterogeneity-preserving alternative; DOT-HF 1.79 vs. OptiLink-HF 0.87). ^2^ Single-trial node; interval widened by the common-τ model. The trial’s native interval is 0.51 (0.38–0.68).

**Table 3:** Acute/in-hospital evidence and gap map.

Modality	Trial	*n*	Primary Endpoint	Result	Hard Outcomes
PA catheter	ESCAPE [45]	433	days alive and out of hospital	neutral (HR 1.00)	death 43 vs. 38; +adverse events
ED lung US	BLUSHED-AHF [46]	130	6 h B-line congestion	neutral (25% vs. 27.5%)	none reported
ReDS at discharge	ReDS-SAFE HF [47]	100	1-month composite	improved (HR 0.094)	HFH 1 vs. 9; death 0 vs. 2
Venous Doppler	VExUS [48]	140	kidney-function recovery	neutral (26% vs. 24%)	90 d death 11 vs. 15; faster decongestion

## Data Availability

All data analyzed in this review were extracted from previously published randomized controlled trials and are presented within the article and its Supplementary Materials. The review protocol is registered with PROSPERO (CRD420261424091). The extraction workbook and analysis code are available from the corresponding author upon reasonable request.

## Supplementary Materials

The following supporting information can be downloaded at: https://www.mdpi.com/article/10.3390/diagnostics16213595/s1, Supplementary File S1: PRISMA 2020 checklist [50]; Supplementary File S2: Search strategies; Table S1: Map of prespecified sensitivity analyses; Table S2: Heterogeneity-estimator and Hartung–Knapp robustness checks; Table S3: Cochrane RoB 2 risk-of-bias assessments; Table S4: CINeMA certainty-of-evidence ratings; Table S5: Transitivity assessment across nodes; and Table S6: trial-level estimand and endpoint map.
