# Supplementary material for: Imaging- and Device-Based Congestion Assessment to Guide Chronic Heart Failure Management: A Systematic Review and Network Meta-Analysis
Source: Diagnostics (Basel). 2026 Oct 30;16(21):3595. doi: 10.3390/diagnostics16213595 (PMC13762208; doi:10.3390/diagnostics16213595)
Supplement: Supplementary file 1 [file diagnostics-16-03595-s001.zip › diagnostics-4543625-supplementary.pdf]

diagnostics

Supplementary Materials

Imaging- and Device-Based Congestion Assessment to Guide Chronic Heart Failure Management: A Systematic Review and Network Meta-Analysis

Contents

Supplementary File S1. PRISMA 2020 Checklist
Supplementary File S2. Search Strategies
Table S1. Map of prespecified sensitivity analyses
Table S2. Heterogeneity-estimator and Hartung–Knapp robustness checks
Table S3. Cochrane RoB 2 risk-of-bias assessments
Table S4. CINeMA certainty-of-evidence ratings
Table S5. Transitivity assessment across nodes
Table S6. Trial-level estimand and endpoint map

Supplementary File S1. PRISMA 2020 Checklist

Supplementary File S1. PRISMA 2020 Checklist. Page numbers refer to the manuscript file submitted to Diagnostics. Rows shaded in amber denote items that are partially reported, with the reason stated in the manuscript.

Section and Topic	Item #	Checklist item	Location where item is reported
**TITLE**			
**Title**	1	Identify the report as a systematic review.	Title page, p. 1: title contains "A Systematic Review and Network Meta-Analysis"
**ABSTRACT**			
**Abstract**	2	Structured summary following the PRISMA 2020 for Abstracts checklist.	Abstract, p. 1: structured as Background/Objectives, Methods, Results, Conclusions
**INTRODUCTION**			
**Rationale**	3	Rationale for the review in the context of existing knowledge.	§1 Introduction, p. 2
**Objectives**	4	Explicit statement of the objective(s) or question(s) addressed.	§1 Introduction, final paragraph, p. 2
**METHODS**			
**Eligibility criteria**	5	Inclusion and exclusion criteria, and how studies were grouped for synthesis.	§2.2 Node-inclusion trigger rule and eligibility, p. 3; §2.3 Excluded classes, p. 4
**Information sources**	6	All databases, registers and other sources searched, with the date each was last searched.	§2.1, p. 3: MEDLINE/PubMed, Embase and Cochrane CENTRAL searched from inception; plus trial registers (ClinicalTrials.gov, WHO ICTRP), reference lists and included-

 https://doi.org/10.3390/diagnostics16213595

Section and Topic	Item #	Checklist item	Location where item is reported
			study tables of prior systematic reviews, and conference proceedings. Searching closed 12 June 2026. Per-source yields in Supplementary File S2, §S2.9
**Search strategy**	7	Full search strategies for all databases and registers, including filters and limits.	Partially reported. Concept blocks and controlled vocabulary for every source: Supplementary File S2, §S2.2–S2.7. The verbatim Boolean strings executed at each database were not retained after completion of the review; noted in §2.1 (p. 3), Supplementary File S2 §S2.9, and §4.4 limitation (7).
**Selection process**	8	Methods used to decide study eligibility, including number of reviewers and independence.	§2.1, p. 3: screened in duplicate against the node-inclusion trigger rule; disagreements resolved by discussion
**Data collection process**	9	Methods used to collect data from reports, including number of reviewers and independence.	§2.5, p. 4: extracted in duplicate by two reviewers, disagreements resolved by discussion; trial investigators were not contacted (Supplementary File S2, §S2.8)
**Data items**	10a	All outcomes for which data were sought.	§2.4 Outcomes, p. 4: primary: HF hospitalization; secondary: all-cause mortality, urgent/unscheduled HF visits, exploratory worsening-HF composite
**Data items**	10b	All other variables for which data were sought; assumptions about missing or unclear information.	§2.5, p. 4; Table 1 (trial characteristics, estimand, follow-up); Table S5 (transitivity variables)
**Study risk of bias assessment**	11	Methods to assess risk of bias, including tool used, number of reviewers and independence.	§2.1, p. 3: Cochrane RoB 2, assessed in duplicate; judgments in Table S3
**Effect measures**	12	Effect measure(s) used for each outcome in synthesis or presentation.	§2.5, p. 4: hazard ratios, recurrent-event rate ratios and risk ratios combined on the log-ratio scale as a single flagged effect measure
**Synthesis methods**	13a	Processes used to decide which studies were eligible for each synthesis.	§2.2 node-inclusion trigger rule, p. 3; §2.6 Model, p. 4
**Synthesis methods**	13b	Methods used to prepare data for synthesis, including handling of missing summary statistics.	§2.5, p. 4: analysis on the log scale with standard errors derived from reported confidence intervals
**Synthesis methods**	13c	Methods used to tabulate or visually display results of individual studies and syntheses.	§3.1–§3.5; Table 1, Table 2; Figures 2–5 (network geometry, forest plots, ranking)
**Synthesis methods**	13d	Methods used to synthesize results, including model, heterogeneity assessment and software.	§2.6 Model, p. 4: contrast-based random-effects NMA, single common τ^2 (DerSimonian–Laird), Bayesian cross-check; Python 3.15.0 (NumPy 2.5.4, SciPy 1.18.1), with an equivalent netmeta + gemtc R script
**Synthesis methods**	13e	Methods used to explore possible causes of heterogeneity.	§2.6, p. 4 (node-specific random-effects and Hartung–Knapp); §2.7, p. 5; Table S2

https://doi.org/10.3390/diagnostics16213595

Section and Topic	Item #	Checklist item	Location where item is reported
**Synthesis methods**	13f	Sensitivity analyses conducted to assess robustness of synthesized results.	§2.7 Prespecified sensitivity analysis plan, p. 5; Table S1 (six prespecified analyses, with outcome applicability)
**Reporting bias assessment**	14	Methods to assess risk of bias due to missing results within a synthesis.	§2.8, p. 5: appraised within the CINeMA reporting-bias domain. Funnel-plot and small-study-effect testing were not feasible (fewer than 10 trials per comparison); stated as a limitation in §4.4
**Certainty assessment**	15	Methods used to assess certainty in the body of evidence for an outcome.	§2.8, p. 5: CINeMA (within-study bias, reporting bias, indirectness, imprecision, heterogeneity; incoherence not assessable in a star network)
		**RESULTS**	
**Study selection**	16a	Results of the search and selection process, ideally using a flow diagram.	§2.1, p. 3 and Figure 1 (PRISMA 2020 flow diagram), p. 7: 3138 records identified; 878 duplicates removed; 2260 screened; 80 full-text reports assessed; 63 excluded with reasons; 17 studies included
**Study selection**	16b	Studies that might appear to meet inclusion criteria but were excluded, and why.	§2.3, p. 4; §3.1, p. 5; Figure 1, p. 7: all 63 full-text exclusions itemized by reason: wrong population/no direct-congestion trigger (22), not randomized (20), natriuretic-peptide-guided (13), duplicate cohort/report (4), CIED multiparameter (3), surrogate endpoint (1)
**Study characteristics**	17	Cite each included study and present its characteristics.	Table 1, p. 6–7; §3.1, p. 5; acute trials in Table 3, p. 13
**Risk of bias in studies**	18	Present risk-of-bias assessments for each included study.	Table S3 (RoB 2 traffic-light table, all 13 chronic trials); summarized §3.7, p. 14
**Results of individual studies**	19	For all outcomes, present each study's effect estimate and precision, ideally using plots.	Table 1, p. 6–7; Figure 3 (HF hospitalization forest plot), p. 10; Figure 5 (mortality forest plot), p. 13
**Results of syntheses**	20a	For each synthesis, summarize characteristics and risk of bias among contributing studies.	§3.2, p. 8; §3.5, p. 11–13; Table 2, p. 9
**Results of syntheses**	20b	Present results of all statistical syntheses, with summary estimate, precision and heterogeneity.	§3.2 and Table 2, p. 8–9; §3.3 ranking, p. 10; §3.5 secondary outcomes, p. 11–13; Figures 3–5
**Results of syntheses**	20c	Present results of all investigations of possible causes of heterogeneity.	§3.4 Sensitivity analyses, p. 11; Table S2 (Paule–Mandel, REML, Hartung–Knapp, Bayesian τ)
**Results of syntheses**	20d	Present results of all sensitivity analyses.	§3.4, p. 11; Table 2 (base case, early-terminated excluded, primary-source-only), p. 9; Table S1
**Reporting biases**	21	Present assessments of risk of bias due to missing results for each synthesis.	§3.7, p. 14; Table S4 (CINeMA reporting-bias domain); limitation stated in §4.4, p. 16

Section and Topic	Item #	Checklist item	Location where item is reported
**Certainty of evidence**	22	Present certainty assessments for each outcome assessed.	§3.7 Certainty of evidence, p. 14; Table S4 (CINeMA ratings per comparison)
		**DISCUSSION**	
**Discussion**	23a	General interpretation of the results in the context of other evidence.	§4 Discussion, p. 14; §4.1 Comparison with prior work, p. 15
**Discussion**	23b	Limitations of the evidence included in the review.	§4.4 Limitations, p. 16
**Discussion**	23c	Limitations of the review processes used.	§4.4 Limitations, p. 16–17: star geometry, mixed estimands, single-trial node, common-τ^2 assumption, and limitation (7): verbatim Boolean strings not retained, so the search is reproducible in substance but not line-for-line; trial-set completeness verified against two independent published syntheses
**Discussion**	23d	Implications for practice, policy and future research.	§4.2 Clinical implications, p. 15; §4.3 Future directions, p. 16; §5 Conclusions, p. 16
		**OTHER INFORMATION**	
**Registration and protocol**	24a	Registration information, including register name and registration number.	§2.1, p. 3: PROSPERO, registered 18 June 2026, registration number CRD420261424091
**Registration and protocol**	24b	Where the review protocol can be accessed.	PROSPERO record: https://www.crd.york.ac.uk/PROSPERO/view/CRD420261424091
**Registration and protocol**	24c	Amendments to information provided at registration or in the protocol.	No amendments were made to the registered protocol. §2.1, p. 3 states the registration date; the protocol was registered during the review rather than before searching, and is described as "registered" rather than prospectively registered.
**Support**	25	Sources of financial and non-financial support, and role of funders.	Funding statement, back matter, p. 17: "This research received no external funding." Consistent with the PROSPERO record (no specific or external funding).
**Competing interests**	26	Competing interests of review authors.	Conflicts of Interest statement, back matter, p. 17: "The authors declare no conflicts of interest." Consistent with the PROSPERO record (no interests declared).
**Availability of data, code and other materials**	27	State which materials are publicly available and where (data, analytic code, forms).	Data Availability Statement, back matter, p. 17: extraction workbook and analysis code (Python, with an equivalent netmeta + gemtc R script) available from the corresponding author upon reasonable request
		**ADDITIONAL ITEMS FOR NETWORK META-ANALYSIS (PRISMA-NMA EXTENSION)**	
**Network geometry: methods**	S1	Describe methods for presenting and summarizing the geometry of the treatment network.	§2.2 node definitions, p. 3; §2.6 Model, p. 4

Section and Topic	Item #	Checklist item	Location where item is reported
**Network geometry: results**	S2	Present the network structure and describe its geometry.	§3.1, p. 5; Figure 2 (network geometry), p. 8: star network, four active nodes versus usual care, no closed loops
**Transitivity**	S3	Describe the assumption of transitivity and how it was appraised.	§2.6, p. 4 (transitivity paragraph); Table S5: appraised across ejection-fraction phenotype, estimand, and signal recipient
**Inconsistency: methods**	S4	Describe statistical methods used to evaluate inconsistency (incoherence).	§2.6, p. 4: the network is a star with no closed loops, so incoherence cannot be statistically evaluated; reported as a structural limitation rather than tested
**Inconsistency: results**	S5	Present results of the inconsistency assessment.	§3.1, p. 5 and §4.4, p. 16: not assessable by design; interpretive consequences discussed

Checklist item wording is summarized for readability; entries correspond to the PRISMA 2020 statement (Page MJ, et al. BMJ 2021;372:n71) and the PRISMA extension for network meta-analyses (Hutton B, et al. Ann Intern Med 2015;162:777–784).

Supplementary File S2. Search Strategies

Search Strategies

Manuscript: Imaging- and Device-Based Congestion Assessment to Guide Chronic Heart Failure Management: A Systematic Review and Network Meta-Analysis. PROSPERO registration CRD420261424091.

All sources searched from inception; searching closed 12 June 2026.

S2.1 Overview and conventions

Three bibliographic databases were searched from inception to 12 June 2026: MEDLINE via PubMed, Embase (Elsevier), and the Cochrane Central Register of Controlled Trials (CENTRAL, Wiley). These were supplemented by screening of trial registers (ClinicalTrials.gov and the WHO ICTRP), hand-searching of reference lists and included-study tables of prior systematic reviews and meta-analyses, and searching of conference proceedings (Section S2.7).

Each database strategy combined the following three concept blocks: (1) heart failure population; (2) imaging- or device-based congestion assessment / monitoring intervention; and (3) a randomized-design filter. CENTRAL indexes controlled trials only and therefore required no design filter. Animal-only records were removed in MEDLINE and Embase.

Conventions: [Mesh] MeSH term; [tiab] title/abstract; [pt] publication type; [ti] title; /exp explode Emtree; :ti,ab,kw title/abstract/keyword; * truncation; * internal wildcard for UK/US spelling variants (h*emodynamic, CENTRAL only).

These term sets record the concept blocks and controlled vocabulary used at each database. The verbatim Boolean strings executed at the time of searching were not retained after completion of the review; the strategies below reproduce the search in substance rather than transcribe the executed queries. Per-source record yields were logged and are reported in Section S2.9.

S2.2 MEDLINE via PubMed

#1 Population

"Heart Failure"[Mesh] OR "heart failure"[tiab] OR "cardiac failure"[tiab] OR "congestive heart failure"[tiab] OR HFrEF[tiab] OR HFpEF[tiab] OR HFmrEF[tiab] OR "Ventricular Dysfunction, Left"[Mesh] OR "cardiac decompensation"[tiab]

#2 Intervention (congestion assessment / monitoring)

"pulmonary artery pressure"[tiab] OR "pulmonary arterial pressure"[tiab] OR CardioMEMS[tiab] OR "left atrial pressure"[tiab] OR HeartPOD[tiab] OR Chronicle[tiab] OR "implantable hemodynamic"[tiab] OR "implantable haemodynamic"[tiab] OR "hemodynamic monitoring"[tiab] OR "haemodynamic monitoring"[tiab] OR "lung ultrasound"[tiab] OR "lung ultrasonography"[tiab] OR "pulmonary ultrasound"[tiab] OR "B-line"[tiab] OR "B-lines"[tiab] OR "B line"[tiab] OR "B lines"[tiab] OR "comet tail"[tiab] OR "lung comet"[tiab] OR "lung comets"[tiab] OR "lung impedance"[tiab] OR "thoracic impedance"[tiab] OR "intrathoracic impedance"[tiab] OR "transthoracic impedance"[tiab] OR OptiVol[tiab] OR "fluid index"[tiab] OR "pulmonary congestion"[tiab] OR "volume overload"[tiab] OR "fluid status"[tiab] OR "fluid overload"[tiab] OR decongest*[tiab] OR "congestion-guided"[tiab] OR "volume-guided"[tiab] OR "remote monitoring"[tiab] OR telemonitoring[tiab] OR "remote patient monitoring"[tiab] OR "Cardiography, Impedance"[Mesh] OR "Monitoring, Ambulatory"[Mesh]

#3 Randomized-design filter (Cochrane Highly Sensitive Search Strategy, sensitivity-maximizing)

"randomized controlled trial"[pt] OR "controlled clinical trial"[pt] OR randomized[tiab] OR randomized[tiab] OR placebo[tiab] OR "clinical trials as topic"[Mesh:NoExp] OR randomly[tiab] OR trial[ti]

#4 Animal exclusion

#3 NOT (animals[Mesh] NOT humans[Mesh])

#5 Final

#1 AND #2 AND #4

Records retrieved: *n* = 1180.

S2.3 Embase (Elsevier, Embase.com)

#1 Population

'heart failure'/exp OR 'heart failure':ti,ab,kw OR 'cardiac failure':ti,ab,kw OR 'congestive heart failure':ti,ab,kw OR HFrEF:ti,ab,kw OR HFpEF:ti,ab,kw OR HFmrEF:ti,ab,kw OR 'heart left ventricle failure'/exp OR 'cardiac decompensation':ti,ab,kw

#2 Intervention

'pulmonary artery pressure':ti,ab,kw OR 'pulmonary arterial pressure':ti,ab,kw OR CardioMEMS:ti,ab,kw OR 'left atrial pressure':ti,ab,kw OR HeartPOD:ti,ab,kw OR Chronicle:ti,ab,kw OR 'implantable hemodynamic':ti,ab,kw OR 'implantable haemodynamic':ti,ab,kw OR 'hemodynamic monitoring':ti,ab,kw OR 'haemodynamic monitoring':ti,ab,kw OR 'lung ultrasound':ti,ab,kw OR 'lung ultrasonography':ti,ab,kw OR 'pulmonary ultrasound':ti,ab,kw OR 'B-line':ti,ab,kw OR 'B-lines':ti,ab,kw OR 'comet tail':ti,ab,kw OR 'lung comet':ti,ab,kw OR 'lung comets':ti,ab,kw OR 'lung impedance':ti,ab,kw OR 'thoracic impedance':ti,ab,kw OR 'intrathoracic impedance':ti,ab,kw OR 'transthoracic impedance':ti,ab,kw OR OptiVol:ti,ab,kw OR 'fluid index':ti,ab,kw OR

'pulmonary congestion':ti,ab,kw OR 'volume overload':ti,ab,kw OR 'fluid status':ti,ab,kw OR 'fluid overload':ti,ab,kw OR decongest*:ti,ab,kw OR 'congestion-guided':ti,ab,kw OR 'volume-guided':ti,ab,kw OR 'remote monitoring':ti,ab,kw OR telemonitoring:ti,ab,kw OR 'remote patient monitoring':ti,ab,kw OR 'thorax impedance'/exp OR 'impedance cardiography'/exp
**#3 Randomized-design filter**
'randomized controlled trial'/exp OR 'controlled clinical trial'/exp OR random*:ti,ab,kw OR placebo:ti,ab,kw OR 'double blind procedure'/exp OR 'single blind procedure'/exp OR (trial:ti)
**#4 Animal exclusion**
#3 NOT ('animal'/exp NOT 'human'/exp)
**#5 Final**
#1 AND #2 AND #4

Records retrieved: *n* = 1540.

S2.4 Cochrane CENTRAL (Wiley Cochrane Library)

CENTRAL indexes controlled trials, so no randomized-design filter is applied; results are limited to Trials.

#1 MeSH descriptor: [Heart Failure] explode all trees<br>#2 ("heart failure" OR "cardiac failure" OR "congestive heart failure" OR HFrEF OR HFpEF OR HFmrEF OR "cardiac decompensation"):ti,ab,kw<br>#3 #1 OR #2<br>#4 ("pulmonary artery pressure" OR "pulmonary arterial pressure" OR CardioMEMS OR "left atrial pressure" OR HeartPOD OR Chronicle OR "implantable h*emodynamic" OR "h*emodynamic monitoring" OR "lung ultrasound" OR "lung ultrasonography" OR "pulmonary ultrasound" OR "B-line*" OR "comet tail" OR "lung comet*" OR "lung impedance" OR "thoracic impedance" OR "intrathoracic impedance" OR "transthoracic impedance" OR OptiVol OR "fluid index" OR "pulmonary congestion" OR "volume overload" OR "fluid status" OR "fluid overload" OR decongest* OR "congestion-guided" OR "volume-guided" OR "remote monitoring" OR telemonitoring OR "remote patient monitoring"):ti,ab,kw<br>#5 MeSH descriptor: [Cardiography, Impedance] this term only<br>#6 #4 OR #5<br>#7 #3 AND #6 (limit: Trials)

Records retrieved (Trials): *n* = 400.

S2.5 ClinicalTrials.gov

Condition/disease: heart failure<br>Intervention/other terms: lung ultrasound OR pulmonary artery pressure OR CardioMEMS OR intrathoracic impedance OR OptiVol OR lung impedance OR congestion OR fluid status<br>Study type: Interventional (clinical trial)<br>Status: All

S2.6 WHO ICTRP

Condition: heart failure<br>Intervention: lung ultrasound OR pulmonary artery pressure OR impedance OR CardioMEMS OR OptiVol OR congestion

S2.7 Supplementary searching

https://doi.org/10.3390/diagnostics16213595

Backward citation searching: reference lists of all included trials and of prior relevant systematic reviews and meta-analyses, including the 2026 lung-ultrasound meta-analysis used for external triangulation.

Forward citation searching: 'cited by' tracking for the index trials of each node (CHAMPION, GUIDE-HF, MONITOR-HF, LUS-HF, IMPEDANCE-HF, and DOT-HF).

Conference proceedings (2018 to 2026): European Society of Cardiology/Heart Failure Association, American College of Cardiology, American Heart Association, and Heart Failure Society of America, for unpublished or interim data reported only in abstract form.

S2.8 Notes and deviations

Spelling variants: CENTRAL (Wiley) uses the internal wildcard h*emodynamic to capture both UK and US spellings; PubMed and Embase list both spellings explicitly because neither platform applies internal wildcards to phrase-searched terms.

Randomized-design filters: PubMed uses the Cochrane Highly Sensitive Search Strategy (sensitivity-maximizing version); Embase uses the equivalent Emtree-based design filter; CENTRAL requires none, as it indexes controlled trials only.

No language restriction was applied at the search stage.

Author contact: trial investigators were not contacted for additional information; all data were extracted from published reports and, where an outcome was unavailable in the primary publication, from a peer-reviewed secondary source with the provenance flagged (Section 2.5 of the main text).

S2.9 Record reconciliation and search documentation

Stage / source	Records	Date searched
MEDLINE via PubMed	1180	12 June 2026
Embase (Elsevier)	1540	12 June 2026
Cochrane CENTRAL (Trials)	400	12 June 2026
**Databases subtotal**	**3120**	**n/a**
Trial registers and hand-searching	18	12 June 2026
**Total records identified**	**3138**	**n/a**
Duplicates removed	878	n/a
Records screened	2260	n/a
Records excluded at title/abstract	2180	n/a
Full-text reports assessed	80	n/a
Full-text reports excluded, with reasons	63	n/a
**Studies included**	**17**	**n/a**

Per-source and per-stage record counts were logged at the time of searching and are reported in the table above and in Figure 1 of the main text. The verbatim Boolean strings executed at each database were not retained after completion of the review, so the search can be reproduced in substance from the term sets above but not line-for-line.

Screening was conducted in duplicate against the prespecified node-inclusion trigger rule. Completeness of the assembled trial set was additionally verified against two independent published syntheses (a meta-analysis of the three pressure-sensor trials and a trial-level meta-analysis of chronic lung-ultrasound trials), neither of which contained an eligible chronic-phase trial absent from this network.

https://doi.org/10.3390/diagnostics16213595

Table S1. Map of prespecified sensitivity analyses.

Sensitivity	Definition	HF hospitalization	All-cause mortality
**S4**	Exclude early-terminated trials (direction-agnostic)	✔	✔
**PS-only**	Primary-source-only; outcome-specific (HFH: −LAPTOP-HF; mortality: −LAPTOP-HF, −CHAMPION)	✔	✔
**GUIDE-HF pre-COVID**	Substitute GUIDE-HF pre-COVID-period HF-hospitalization estimate	✔	*N/A (by design)* [a]
**EPICC mortality**	Exclude EPICC (ADHF-attributable death only) from the mortality pool	*N/A (by design)* [b]	✔
**S3 within-class split**	Split N1 into PA / LA / RV pressure subtypes (exploratory)	**✔ (exploratory)**	n/a
**Estimand-restricted**	Restrict N1 to its recurrent-event trials, excluding the composite-derived REDUCEhf	✔	*N/A (by design)* [c]
**S5 fixed-effect**	Fixed-effect re-estimation (model-assumption check)	✔	✔

[a] No pre-COVID mortality input exists: the COVID-period subset was prespecified for HF hospitalization only (Lindenfeld 2021). [b] EPICC's HF-hospitalization contribution is already removed by S4; a separate HFH-EPICC-excluded run would be post hoc and is not prespecified. [c] The restriction addresses the mixture of HF-hospitalization estimands and has no mortality counterpart. PS-only, primary-source-only; ADHF, acute decompensated heart failure; PA/LA/RV, pulmonary-artery/left-atrial/right-ventricular. "n/a" in the within-class split row, not applied.

Table S2. Heterogeneity-estimator and Hartung–Knapp robustness checks.

Strategy node	DL (primary)	Paule–Mandel	REML	Hartung–Knapp
**N3 Lung impedance**	0.51 (0.31–0.83)	0.51 (0.28–0.91)	0.51 (0.30–0.86)	n/a (single trial)
**N1 Pressure**	0.72 (0.57–0.90)	0.72 (0.55–0.93)	0.72 (0.56–0.91)	0.72 (0.56–0.93)
**N2 Lung US**	0.72 (0.48–1.08)	0.73 (0.47–1.12)	0.72 (0.48–1.09)	0.73 (0.38–1.40)
**N5 CIED impedance**	1.12 (0.77–1.63)	1.15 (0.74–1.79)	1.13 (0.76–1.69)	1.21 (0.01–115.8) ‡
Common τ^2 (τ)	*0.040 (0.199)*	*0.066 (0.258)*	*0.049 (0.221)*	*n/a*

All values are HF-hospitalization effect (95% CI) versus usual care under the common-τ model; point estimates are invariant to the estimator. DL, DerSimonian–Laird. Hartung–Knapp uses node-specific between-trial variance and a t-distribution with k−1 degrees of freedom; not estimable for single-trial nodes. ‡ With only two opposite-direction trials, the Hartung–Knapp interval for N5 is uninformative, evidence that this node cannot be estimated reliably. Bayesian τ posterior median 0.25 (95% credible interval 0.03–0.66).

Table S3. Cochrane RoB 2 risk-of-bias assessments.

Trial	D1	D2	D3	D4	D5	Overall
**N1 Pressure**						
CHAMPION	+	?	+	+	+	?
GUIDE-HF	+	?	+	+	?	?
MONITOR-HF	+	?	+	+	+	?
LAPTOP-HF	+	?	?	?	–	–
REDUCEhf	+	?	?	?	–	–
**N2 Lung US**						
LUS-HF	+	?	+	?	+	?
Marini	+	?	?	?	–	–

**Trial**	**D1**	**D2**	**D3**	**D4**	**D5**	**Overall**
CLUSTER-HF	+	?	?	?	?	?
Zisis	?	?	?	–	?	–
EPICC	?	?	?	–	?	–
			**N3 Lung impedance**			
IMPEDANCE-HF	+	?	+	?	+	?
			**N5 CIED impedance**			
DOT-HF	+	–	+	–	–	–
OptiLink-HF	+	?	+	+	+	?

Key: + Low ? Some concerns – High. RoB 2 domain and overall judgments were made independently in duplicate by two reviewers, with disagreements resolved by discussion, against the design and reporting features of each trial, including open-label execution, early termination, provenance of the extracted estimate, endpoint definition, and completeness of outcome reporting. D1, randomization process; D2, deviations from intended interventions; D3, missing outcome data; D4, measurement of the outcome; D5, selection of the reported result.

Table S4. CINeMA certainty-of-evidence ratings.

**Comparison (vs usual care)**	**WSB**	**RB**	**Ind**	**Imp**	**Het**	**Inc**	**Confidence**
**N1 Pressure**	?	?	+	+	?	*n/a*	**Moderate**
**N3 Lung impedance**	?	+	?	✗	*n/a*	*n/a*	**Low**
**N2 Lung US**	✗	?	?	✗	✗	*n/a*	**Very low**
**N5 CIED impedance**	✗	?	?	✗	✗	*n/a*	**Very low**

Key: + no concerns ? some concerns ✗ major concerns. CINeMA confidence ratings for the HF-hospitalization comparisons; within-study bias is inherited from Table S3. WSB, within-study bias; RB, reporting bias; Ind, indirectness; Imp, imprecision; Het, heterogeneity; Inc, incoherence (not evaluable in a star network with no closed loops). Heterogeneity is not estimable for the single-trial N3 node. Confidence: High > Moderate > Low > Very low.

Table S5. Transitivity assessment across nodes.

**Trial**	**EF / phenotype**	**NYHA**	**Setting / follow-up**	**Trigger**	**Signal recipient**	**HFH outcome**
			N1 Pressure			
CHAMPION	No EF criterion	III	Ambulatory; prior HF admission (≤12 mo); 6-mo randomized period analyzed	PA pressure	Clinician	Recurrent
GUIDE-HF	No EF criterion	II–IV	Ambulatory; prior HFH ≤12 mo or elevated NP; 12 mo	PA pressure	Clinician	Recurrent
MONITOR-HF	No EF criterion	III	Ambulatory; prior HF admission ≤12 mo; mean 1.8 y	PA pressure	Clinician	Recurrent
LAPTOP-HF	No EF criterion	III	Ambulatory; prior HFH ≤12 mo or elevated NP; 12 mo; stopped early (DSMB, implant complications)	LA pressure	Clinician + patient	Recurrent
REDUCEhf	HFrEF (mean LVEF 23%)	II–III	Ambulatory; prior HFH ≤12 mo; ICD indication; 12 mo;	RV pressure	Clinician	Composite

Trial	EF / phenotype	NYHA	Setting / follow-up	Trigger	Signal recipient	HFH outcome
			stopped early (sensor-lead failures)			
			N2 Lung US			
LUS-HF	No EF criterion (mean LVEF 39%)	Not specified	Randomized at discharge after an HF admission; 6 mo	Lung US	Clinician	First
Marini	LVEF <45%	Not specified	Chronic ambulatory; HF ≥6 mo; 3 mo; stopped early for benefit	Lung US	Clinician	First
CLUSTER-HF	No EF criterion (median LVEF 31%)	Not specified	Randomized at discharge after an HF admission; 6 mo	Lung US	Clinician	Composite
Zisis	No EF criterion (HFrEF/HFpEF strata)	Not specified	Post-emergency-admission, high-risk only; 3 mo; 122 of 404 planned randomized	Lung US	Clinician	First
EPICC	No EF criterion (52% with LVEF >50%)	≥II	Selected within 36 h of discharge; mean age 81 y; 6 mo; stopped early at interim analysis	Lung US	Clinician	First
			N3 Lung impedance			
IMPEDANCE-HF	LVEF ≤35%	II–IV	Ambulatory post-discharge; AHF admission ≤12 mo; 12-mo window analyzed (total follow-up 48 vs 39 mo)	Lung impedance (TTI)	Clinician	First
			N5 CIED impedance			
DOT-HF	No EF criterion stated; ICD 18% / CRT-D 82%	II–IV	Existing CIED; HFH or urgent visit ≤12 mo; mean 14.9 mo; stopped early (slow enrollment, futility)	CIED impedance	Patient (audible)	First
OptiLink-HF	LVEF ≤35%	II–III	Enrolled 3–21 d after CIED implant; mean 1.9 y	CIED impedance	Physician (silent)	Recurrent

Eligibility and follow-up values are taken from the primary trial reports, supplemented where needed by the trial registry record or published design paper. "Not specified" means the trial stated no eligibility criterion for that characteristic. Intervention trigger, signal recipient, and HF-hospitalization outcome definition are taken from the trial designs and the locked estimand tags (Table S6). PA, pulmonary-artery; LA, left-atrial; RV, right-ventricular; TTI, transthoracic impedance; CIED, cardiac implantable electronic device; ICD, implantable cardioverter-defibrillator; CRT-D, cardiac resynchronization therapy defibrillator; HFH, HF hospitalization; NP, natriuretic peptide. Transitivity is most defensible for the pressure node, in which four of the five trials enrolled irrespective of ejection fraction (REDUCEhf, which required an ICD indication, enrolled reduced ejection fraction only) under clinician-titrated protocols, and least defensible for the CIED-impedance node, which is available only to patients already carrying a device implanted for another indication and in which the two trials differ in whether the fluid alert reaches the patient or the physician. The lung-ultrasound node is intermediate: its trials are small and open-label, and most did not specify eligibility by ejection fraction or NYHA class.

https://doi.org/10.3390/diagnostics16213595

Table S6. Trial-level estimand and endpoint map.

Trial [ref]	Node	*n* (int/ctrl)	HF-hospitalization endpoint as analyzed	Statistical estimand	Analysis window	Effect (95% CI)	Event structure and provenance
CHAMPION [3,4]	N1 Pressure	270/280	Recurrent HF hospitalizations	Recurrent-event	6 mo	0.72 (0.60–0.85)	Rate over the randomized single-blind access period
GUIDE-HF [5]	N1 Pressure	497/503	Recurrent HF hospitalizations	Recurrent-event	12 mo	0.83 (0.68–1.01)	Overall ITT base case; prespecified pre-COVID analysis 0.72 (sensitivity analysis)
MONITOR-HF [6]	N1 Pressure	176/172	Recurrent HF hospitalizations (pure HF-hospitalization endpoint)	Recurrent-event	22 mo	0.56 (0.38–0.82)	Pure HF-hospitalization endpoint; no composite decomposition required
LAPTOP-HF [7,8]	N1 Pressure	~243/243	Recurrent HF hospitalizations	Recurrent-event	24 mo	0.57 (0.40–0.83)	Early-terminated (implant-related safety); interval from secondary source, flagged provenance
REDUCEhf [9]	N1 Pressure	202/198	HF hospitalization derived from a reported composite	Composite-derived	12 mo	0.99 (0.61–1.61)	Early-terminated; HF-hospitalization component not reported in isolation
LUS-HF [10]	N2 Lung US	61/62	First HF hospitalization	First-event	6 mo	1.10 (0.51–2.35)	Null result
Marini [11]	N2 Lung US	127/117	First HF hospitalization	First-event	3 mo	0.44 (0.23–0.84)	Stopped early for benefit; principal contributor to the S4 fragility analysis
CLUSTER-HF [12]	N2 Lung US	63/63	HF hospitalization derived from a reported composite	Composite-derived	6 mo	0.50 (0.15–1.60)	Sparse: 12 events in total
Zisis [13]	N2 Lung US	58/64	First HF readmission within 90 days	First-event	3 mo	0.72 (0.39–1.31)	High risk of bias; early-terminated (122 of 404 planned randomized); readmission window shorter than other node members
EPICC [14]	N2 Lung US	37/42	First HF hospitalization	First-event	6 mo	1.90 (0.53–7.06)	Not significant; early-terminated at interim analysis; mortality endpoint captured ADHF-attributable death only
IMPEDANCE-HF [15]	N3 Lung impedance	128/128	First HF hospitalization	First-event	12 mo	0.51 (0.38–0.68)	Single-trial node; 48-month total trial follow-up, 12-month analysis window extracted
DOT-HF [16]	N5 CIED impedance	168/167	First HF hospitalization	First-event	15 mo	1.79 (1.08–2.95)	Audible patient-facing alert; early-terminated
OptiLink-HF [17]	N5 CIED impedance	505/497	Recurrent HF hospitalizations	Recurrent-event	23 mo	0.87 (0.67–1.12)	Silent physician-facing alert

Estimand classes follow the extraction recorded in Table 1 of the main text. Trial-level contrasts were combined on a common log-ratio scale as described in Section 2.5; because first-event, recurrent-event, and composite-derived endpoints do not estimate an identical underlying quantity, pooled node estimates are reported as ratio measures rather than as a single homogeneous HF-hospitalization effect. The prespecified estimand-restricted sensitivity analysis (Table S1) tests this harmonization within the pressure node, where recurrent-event counting predominates. Analysis window is the follow-up period from which the HF-hospitalization estimate was extracted, which may be shorter than total trial follow-up.

https://doi.org/10.3390/diagnostics16213595
